# Synthesis and crystal structure of bis­[μ_2_-7-({bis­[(pyridin-2-yl)meth­yl]amino-κ^3^*N*,*N*′,*N*′′}meth­yl)-5-chloro­quinolin-8-olato-κ^2^*N*,*O*]dizinc(II) bis­(perchlorate) aceto­nitrile monosolvate

**DOI:** 10.1107/S2056989025010680

**Published:** 2026-01-01

**Authors:** Koji Kubono, Keita Tani, Yukiyasu Kashiwagi

**Affiliations:** a Osaka Kyoiku University, 4-698-1 Asahigaoka Kashiwara Osaka 582-8582 Japan; b Osaka Research Institute of Industrial Science and Technology, 1-6-50 Morinomiya Joto-ku Osaka 536-8553 Japan; Tokyo University of Science, Japan

**Keywords:** crystal structure, zinc(II) complex, dimeric dinuclear structure, 8-quinolinol, bis­(2-picoly)amine, C—H⋯O inter­actions

## Abstract

The title compound, [Zn_2_(C_22_H_18_ClN_4_O)_2_](ClO_4_)_2_·CH_3_CN, consists of one centrosymmetric cationic dinuclear zinc(II) complex with two penta­dentate ligands containing quinolin-8-olato and bis­(pyridin-2-ylmeth­yl)amine groups, two perchlorate counter-ions and one aceto­nitrile solvate mol­ecule. The Zn^II^ atom adopts a distorted octa­hedral geometry and coordinates the O atom and the N atom of the quinolin-8-olato group and three N atoms of the bis­(pyridin-2-ylmeth­yl)amine group in a ligand, and the O atom in an adjacent ligand generated by an inversion operation. The phenolato oxygen atoms in the two ligands of the cationic dinuclear complex are bridging coordinated with the two Zn^II^ atoms. In the crystal, the cationic dinuclear complex mol­ecules and perchlorate ions are linked by C—H⋯Cl and C—H⋯O hydrogen bonds, forming a three-dimensional network.

## Abstract 2

The title compound is a centrosymmetric cationic dinuclear zinc(II) complex with two penta­dentate ligand containing quinolin-8-olato and bis­(pyridin-2-ylmeth­yl)amine groups, two perchlorate counter-ions and one aceto­nitrile solvate mol­ecule. The Zn^II^ atom adopts a distorted octa­hedral geometry and coordinates the O atom and the N atom of the quinolin-8-olato group and three N atom of the bis­(pyridin-2-ylmeth­yl)amine group in a ligand, and the O atom in an adjacent ligand generated by an inversion operation. In the crystal, the cationic dinuclear complex mol­ecules and perchlorates are linked by C—H⋯Cl and C—H⋯O hydrogen bonds to form a three-dimensional network.

## 1. Chemical context

Dinuclear metal complexes have recently gained considerable attention due to their applications in various fields, including catalysis (Ouyang *et al.*, 2018 ▸), magnetic materials (Rabelo *et al.*, 2020 ▸) and biosensors (Das & Gupta, 2021 ▸). Dinuclear metal complex with quinolin-8-ol (Hq) derivatives have wide applications in diverse areas such as magnetic and luminescent materials (Shen *et al.*, 2015 ▸; Wang *et al.*, 2016 ▸). We synthesized a penta­dentate ligand (HClqdpa) containing Hq and bis­(pyridin-2-ylmeth­yl)amine [di-(2-picol­yl)amine, dpa] moieties (Kubono *et al.*, 2015 ▸). HClqdpa forms a mononuclear Zn:ligand = 1:1 complex with zinc(II) bromide, ZnBr_2_(HClqdpa), and a dinuclear Zn:ligand = 2:1 complex with zinc(II) chloride, Zn_2_Cl_3_(Clqdpa) (Kubono *et al.*, 2022 ▸, 2024 ▸). These Zn^II^ complexes contain strongly donating anions, but a zinc(II) salt with a weakly donating anion can form a complex with a different structure with the ligand.
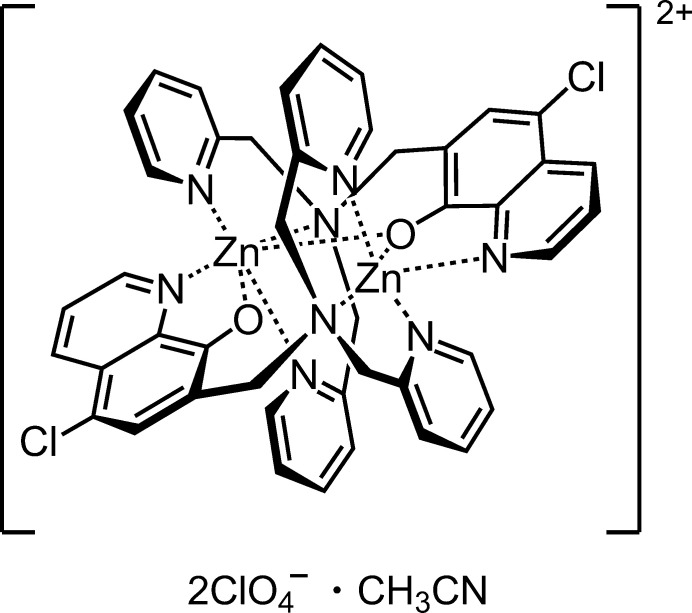


Herein we report on the synthesis of a dimeric dinuclear ion-pair Zn:ligand = 2:2 complex between zinc(II) perchlorate and HClqdpa, [Zn_2_(Clqdpa)_2_](ClO_4_)_2_, and crystal structure of its aceto­nitrile solvate.

## 2. Structural commentary

**Figure 1:**
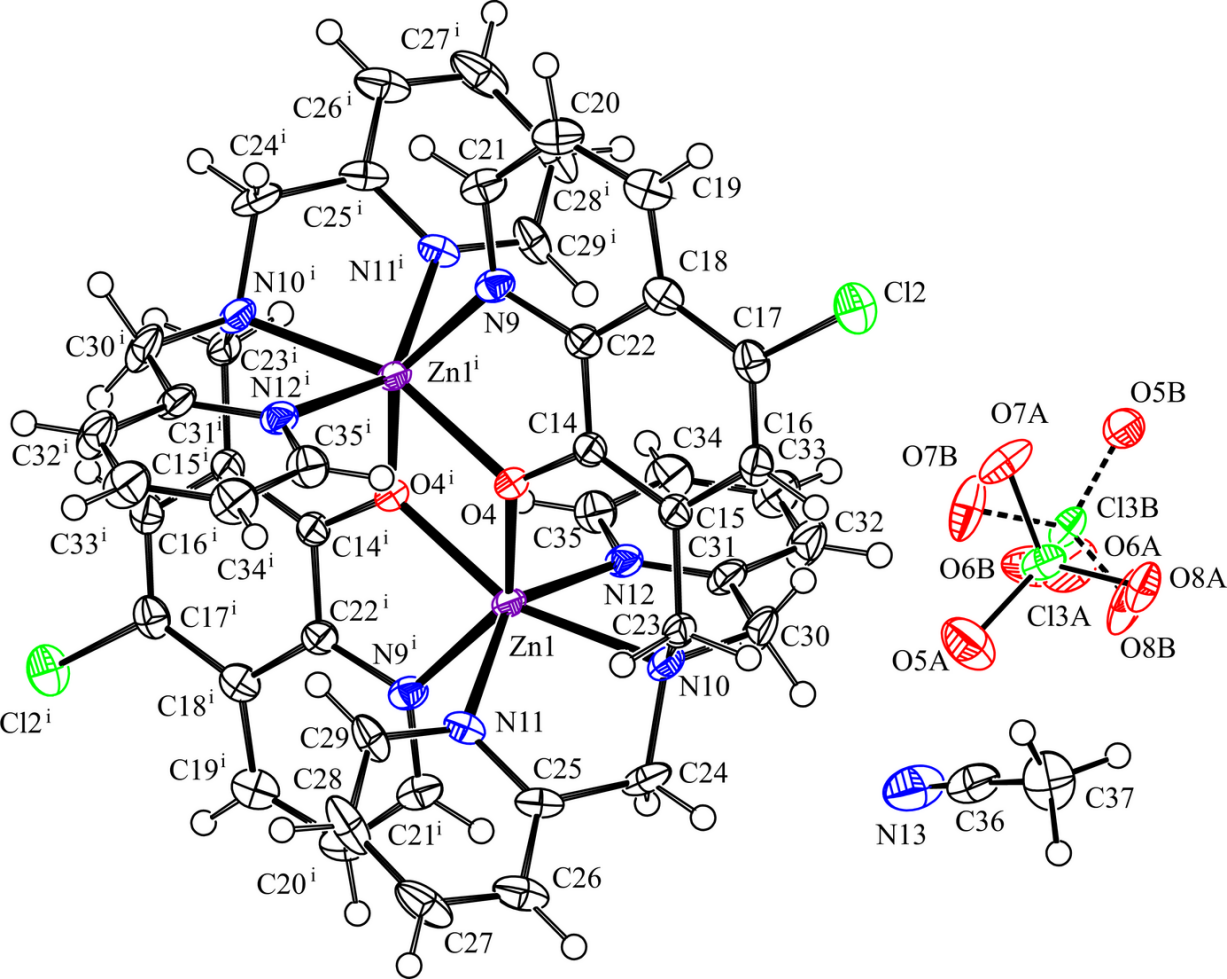
The mol­ecular structure of the title compound, with the atom labelling. Displacement ellipsoids are drawn at the 50% probability level. The major occupancy perchlorate ion is drawn using unbroken lines (*A*) and the minor disorder component is drawn using dashed lines (*B*). H atoms are represented by spheres of arbitrary radius. [Symmetry code: (i) −*x* + 1, −*y* + 1, −*z* + 1.]

The mol­ecular structure of the title compound is shown in Fig. 1 ▸. The asymmetric unit is composed of one Zn^II^ atom, one Clqdpa ligand, one perchlorate anion and one-half of an aceto­nitrile solvate mol­ecule. The solvate mol­ecule is disordered within the cavities around a centre of inversion, which is located in the middle of the methyl groups of the two aceto­nitrile mol­ecules. The Zn^II^ complex is a centrosymmetric dinuclear structure. The Zn1 atom adopts a distorted octa­hedral geometry and coordinates the O atom of the quinolinol unit and three N atoms of the dpa unit in one Clqdpa ligand, and the O atom and the N atom in an adjacent Clqdpa ligand generated by the inversion operation. The phenolato oxygen atoms in the two ligands of the dinuclear complex are bridging coordinated with the two Zn^II^ atoms. The Zn1—O4 bond distance is 2.0496 (13) Å, shorter than that the of Zn1—O4^i^ [2.0906 (12) Å; symmetry code: (i) 1 − *x*, 1 − *y*, 1 − *z*] (Table 1 ▸). The Zn1—N10 (aliphatic tertiary amine) is 2.3072 (16) Å, longer than those of the Zn—N (aromatic amine) [Zn1—N9^i^, Zn1—N11, Zn1—N12 are 2.2527 (16), 2.1171 (15) and 2.0868 (15) Å, respectively]. The parameter σ, proposed by Zhu *et al.* (2008 ▸) to qu­antify the degree of distortion of an octa­hedral geometry, is 0.592, indicating a substantial distortion. This angular structural parameter, defined as *σ* = [*α*_min_ + *α*_max_ − 180]/90, is evaluated from the minimum angle and maximum angle (*α*_min_, *α*_max_), and has a value of 1 for an ideal octa­hedral geometry. The related polymorphs of the Zn^II^ complexes with a ligand in which the Cl atom of HClqdpa is replaced with an H atom, bis­(μ-7-({bis­[(pyridin-2-yl)meth­yl]amino}­meth­yl)quinolin-8-olato)di­zinc(II) bis­(tetra­phenyl­borate), [Zn_2_(qdpa)_2_](BPh_4_)_2_ have *σ* parameters of 0.582 (for the *P*2_1_/*c* polymorph) and 0.426 (for the *P*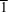 polymorph) (CSD refcodes FEDTUH and FEDTOB; Kong *et al.*, 2022 ▸). The Zn1⋯Zn1^i^ distance within the dinuclear complex is 3.2829 (3) Å, similar to those of the related Zn^II^ complexes (3.231 Å for FEDTUH and 3.247 Å for FEDTOB). The Zn1—O4—Zn1^i^ angle is 104.92 (5)° (Table 1 ▸), which is close to 102.34 (6)° for FEDTUH and 103.28 (5)° for FEDTOB. The other related complex with the same combination of ligand skeleton and substituents is bis­(μ_2_-[bis­(2-pyridyl­meth­yl)-8-(­oxy)quinoline-2-meth­yl]amine)­dizinc(II) diperchlorate (RIZROI; Xue *et al.*, 2008 ▸). In this complex, the Zn⋯Zn distance is 3.496 Å and the Zn—O—Zn angle is 109.71 (17)°, and the σ parameter is 0.402. In the related Zn:ligand 2:1 complex between HClqdpa and zinc(II) chloride, Zn_2_Cl_3_(Clqdpa), in which the Zn^II^ atoms adopt a tetra­hedral and a distorted trigonal–bipyramidal geometry, the Zn⋯Zn distance is 3.3684 (9) Å and the Zn—O—Zn angle is 112.72 (12)° (Kubono *et al.*;,2024 ▸).

## 3. Supra­molecular features

**Figure 2:**
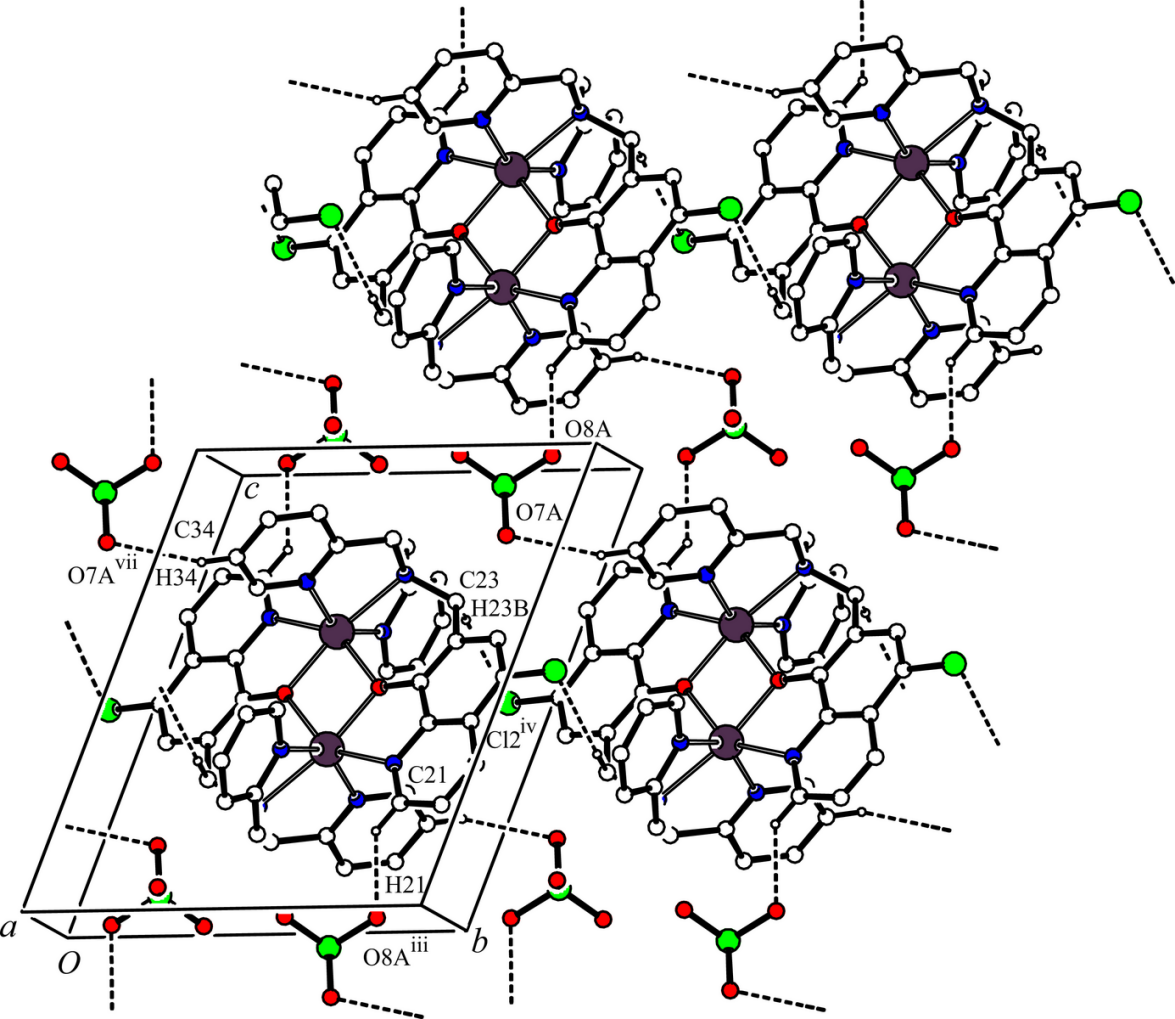
Two-dimensional network structure between [Zn_2_(Clqdpa)_2_]^2+^ and the major occupancy component of the perchlorate ion parallel to the *bc* plane. The inter­molecular C21—H21⋯O8*A*^iii^, C23—H23*B*⋯Cl2^iv^ and C34—H34⋯O7*A*^vii^ hydrogen bonds are shown as dashed lines. H atoms not involved in the inter­actions and all components of the aceto­nitrile solvate mol­ecule have been omitted for clarity. [Symmetry codes: (iii) *x*, *y*, *z* − 1; (iv) −*x* + 1, −*y* + 2, −*z* + 1; (vii) *x*, *y* − 1, *z*.]

**Figure 3:**
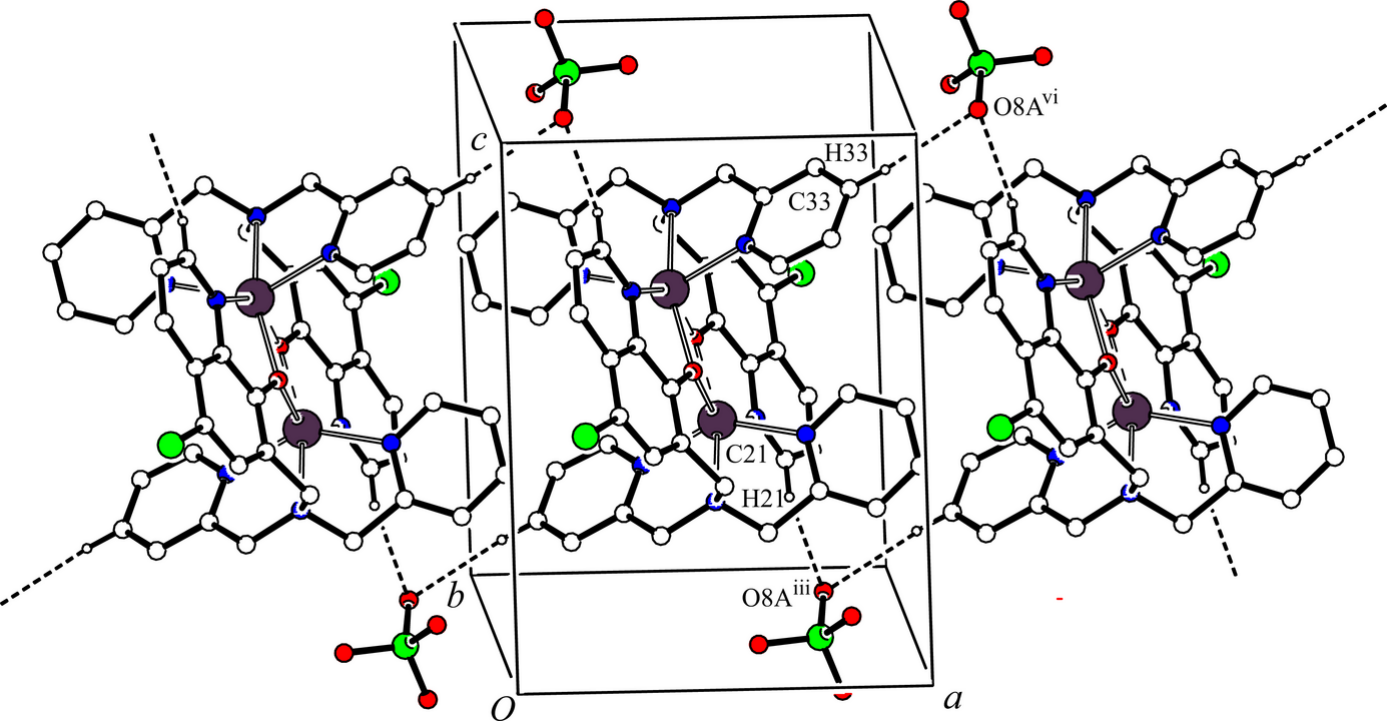
One-dimensional network structure between [Zn_2_(Clqdpa)_2_]^2+^ and the minor occupancy component of the perchlorate ion along the *a-*axis direction. The inter­molecular C21—H21⋯O8*A*^iii^ and C33—H33⋯O8*A*^vi^ hydrogen bonds are shown as dashed lines. H atoms not involved in the inter­actions and all components of the aceto­nitrile solvate mol­ecule have been omitted for clarity. [Symmetry codes: (iii) *x*, *y*, *z* − 1; (vi) −*x* + 2, −*y* + 1, −*z* + 2.]

In the crystal, two cationic dinuclear complex mol­ecules are associated through a pair of inter­molecular C—H⋯Cl hydrogen bonds [C23—H23B⋯Cl2^iv^; symmetry code: (iv) 1 − *x*, 2 − *y*, 1 − *z*; Table 2 ▸] and an inversion operation, forming a dimer with an 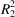
(12) ring motif and a one-dimensional network propagating along the *b-*axis direction. Another one-dimensional network is generated by inter­molecular C—H⋯O hydrogen bonds between the cationic dinuclear complex and the major occupancy perchlorate ion [C21—H21⋯O8*A*^iii^ and C34—H34⋯O7*A*^vii^; symmetry codes: (iii) *x*, *y*, *z* – 1; (vii) *x*, *y* – 1, *z*] (Table 2 ▸) along the [0
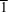
1] direction. These inter­molecular C—H⋯Cl and C—H⋯O hydrogen bonds generate a two-dimensional network lying parallel to the *bc* plane (Fig. 2 ▸). Furthermore, there are other inter­molecular C—H⋯O hydrogen bonds between the cationic dinuclear complex and the major occupancy component of the perchlorate ion [C21—H21⋯O*8A*^iii^ and C33—H33⋯O8*A*^vi^; symmetry codes: (iii) *x*, *y*, *z* – 1; (vi) 2 − *x*, 1 − *y*, 2 − *z*] (Table 2 ▸), forming a one-dimensional network along the *a-*axis direction (Fig. 3 ▸). In the crystal, the cationic dinuclear complex mol­ecules and major occupancy perchlorate ions are linked by inter­molecular C—H⋯Cl and C—H⋯O hydrogen bonds, forming a three-dimensional network structure.

**Figure 4:**
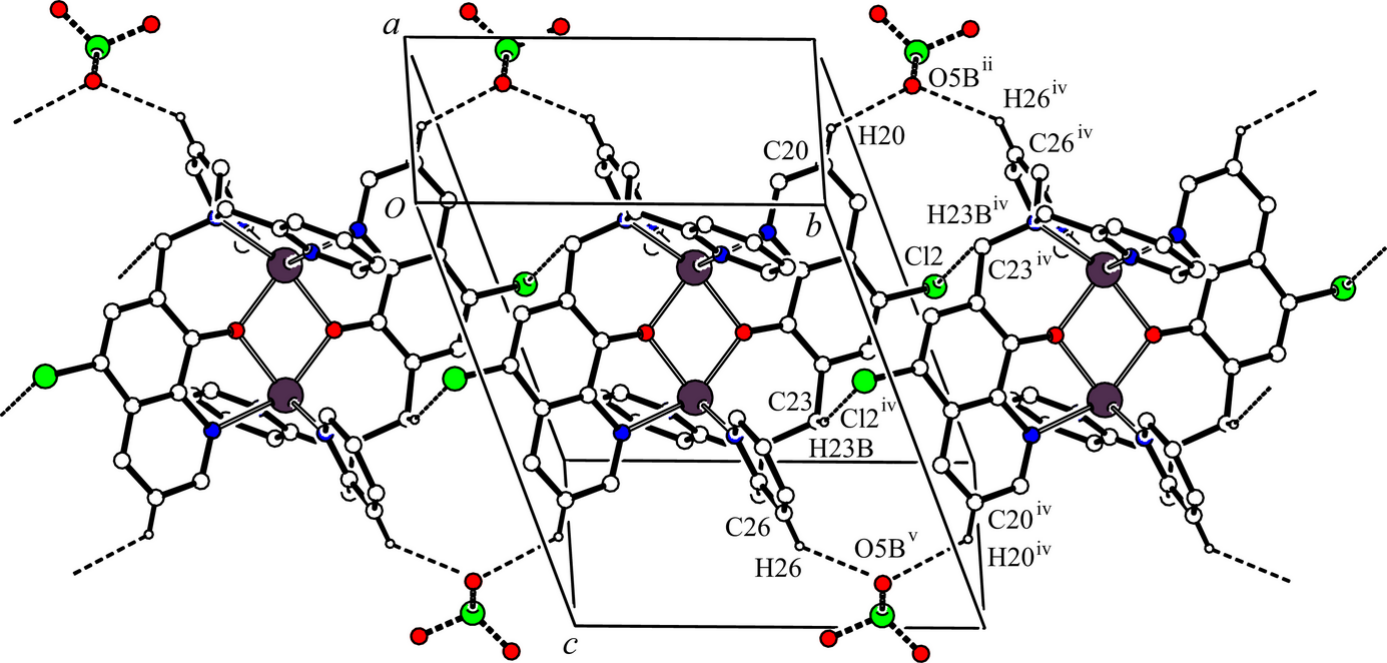
One-dimensional network structure between [Zn_2_(Clqdpa)_2_]^2+^ and the minor occupancy perchlorate ion along the *b*-axis direction. The inter­molecular C20—H20⋯O5*B*^ii^, C23—H23*B*⋯Cl2^iv^ and C26—H26⋯O5*B*^v^ hydrogen bonds are shown as dashed lines. H atoms not involved in the inter­actions have been omitted for clarity. [Symmetry codes: (ii) −*x* + 2, −*y* + 2, −*z* + 1; (iv) –*x* + 1, –*y* + 2, –*z* + 1; (v) *x* − 1, *y*, *z*.]

**Figure 5:**
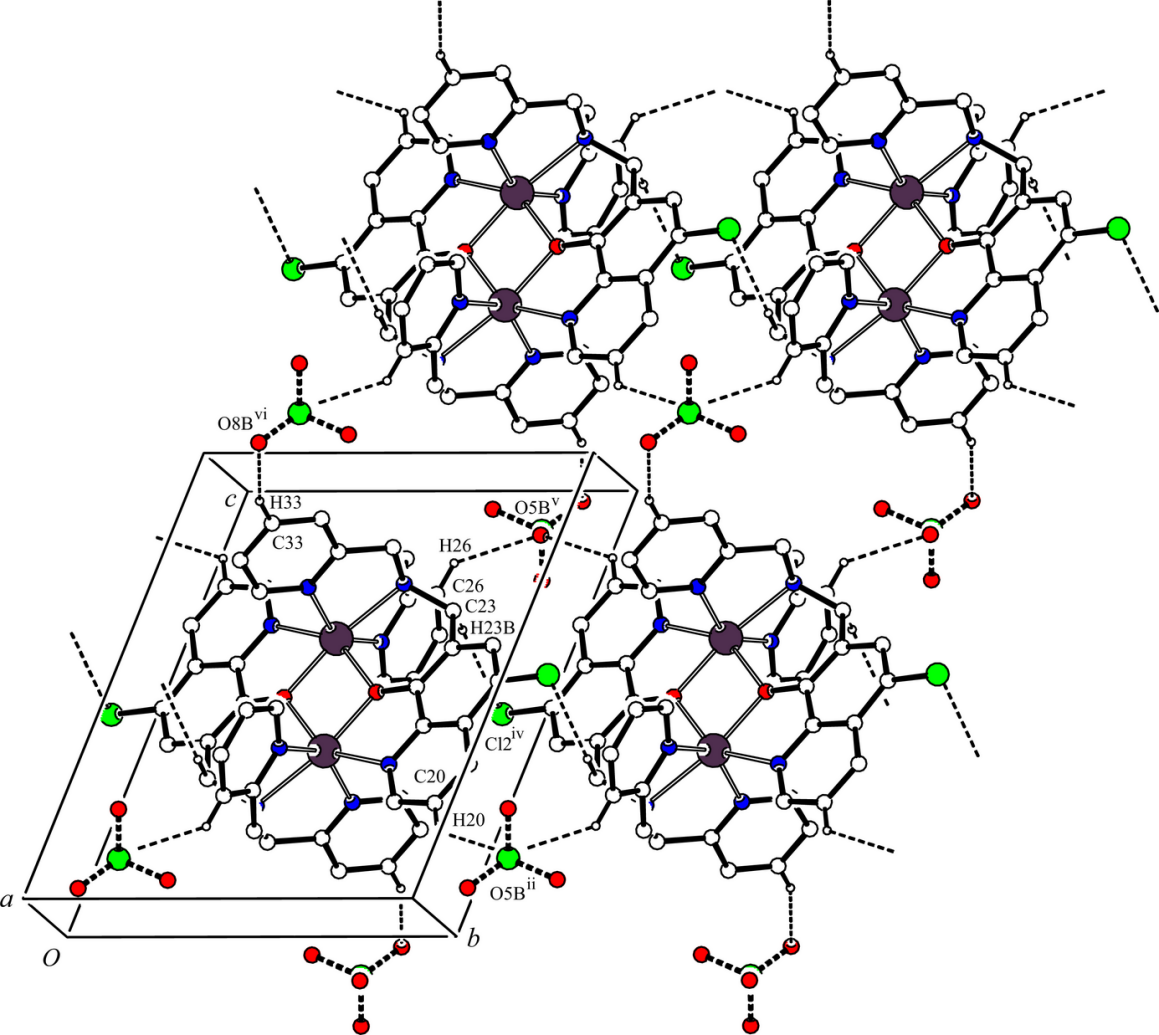
Two-dimensional network structure between [Zn_2_(Clqdpa)_2_]^2+^ and the minor occupancy perchlorate ion parallel to the *bc* plane. The inter­molecular C20—H20⋯O5*B*^ii^, C23—H23⋯Cl2^iv^, C26—H26⋯O5*B*^v^ and C33—H33⋯O8*B*^vi^ hydrogen bonds are shown as dashed lines. H atoms not involved in the inter­actions and all components of the aceto­nitrile solvate mol­ecule have been omitted for clarity. [Symmetry codes: (ii) −*x* + 2, −*y* + 2, −*z* + 1; (iv) −*x* + 1, −*y* + 2, −*z* + 1; (v) *x* − 1, *y*, *z*; (vi) −*x* + 2, −*y* + 1, −*z* + 2.]

**Figure 6:**
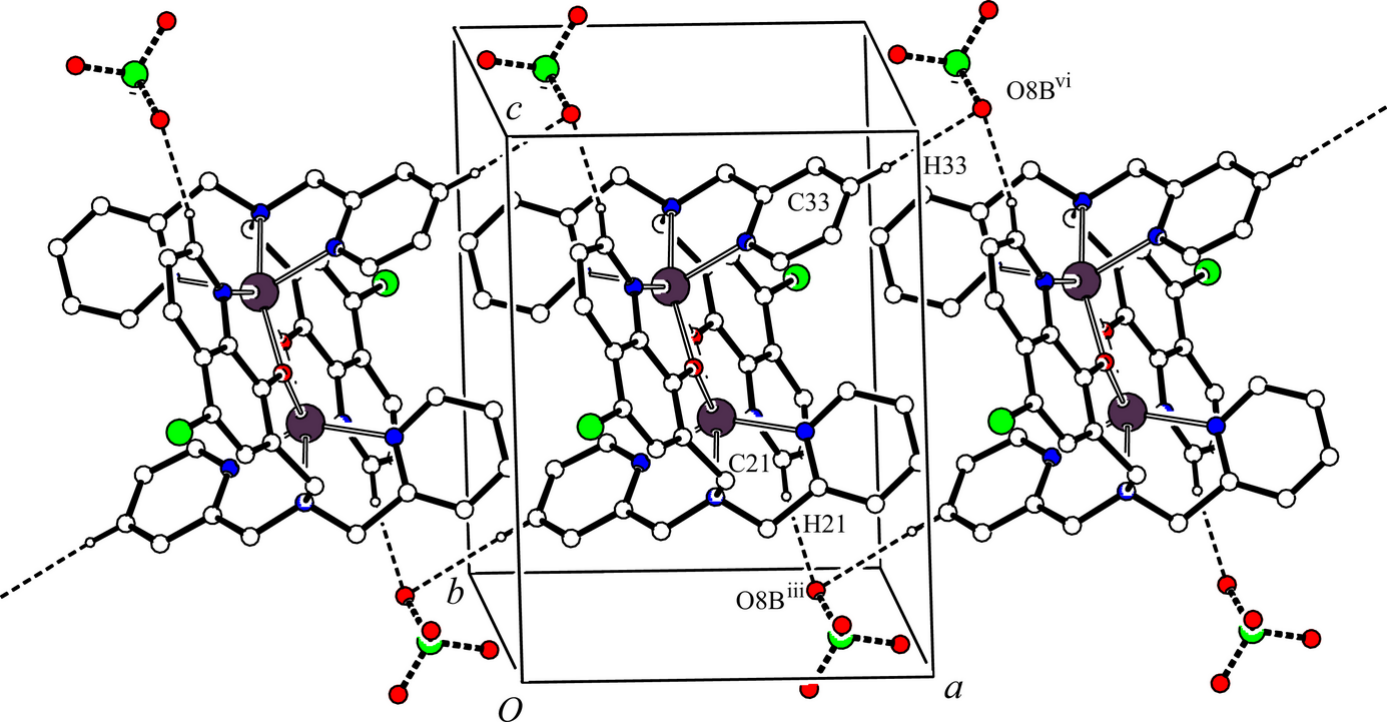
Another one-dimensional network structure between [Zn_2_(Clqdpa)_2_]^2+^ and the minor occupancy perchlorate ion along the *a-*axis direction.. The inter­molecular C21—H21⋯O8*B*^iii^ and C33—H33⋯O8*B*^vi^ hydrogen bonds are shown as dashed lines. H atoms not involved in the inter­actions have been omitted for clarity. [Symmetry codes: (iii) *x*, *y*, *z* − 1; (vi) −*x* + 2, −*y* + 1, −*z* + 2.]

The minor occupancy perchlorate ion also forms network structures with the cationic dinuclear complex mol­ecule, similar to that of between its major disorder component and the dinuclear complex. In the crystal, there are inter­molecular C—H⋯O hydrogen bonds between the cationic dinuclear complex and the minor occupancy perchlorate ion [C20—H20⋯O8*B*^ii^, C23—H23*B*⋯Cl2^iv^ and C26—H26⋯O5*B*^v^; symmetry codes: (ii) 2 − *x*, 2 − *y*, 1 − *z*; (iv) 1 − *x*, 2 − *y*, 1 − *z*; (v) *x* – 1, *y*, *z*] (Table 2 ▸), forming a one-dimensional network along the *b-*axis direction (Fig. 4 ▸). These inter­molecular hydrogen bonds and C33—H33⋯O8*B*^vi^ hydrogen bonds [symmetry code: (vi) 2 − *x*, 1 − *y*, 2 − *z*] (Table 2 ▸) generate a two-dimensional network parallel to the *bc* plane (Fig. 5 ▸). Another one-dimensional network is formed by inter­molecular C—H⋯O hydrogen bonds [C21—H21⋯O8*B*^iii^ and C33—H33⋯O8*B*^vi^; symmetry codes (iii) *x*, *y*, *z* – 1; (vi) 2 − *x*, 1 − *y*, 2 − *z* (Table 2 ▸)] along the *a-*axis direction (Fig. 6 ▸). In the crystal, the dinuclear complex mol­ecules and the minor occupancy perchlorate ions are also linked by C—H⋯Cl and C—H⋯O hydrogen bonds, forming a three-dimensional network structure.

## 4. Database survey

A search of the Cambridge Structural Database (CSD, 6.00, update of August 2025; Groom *et al.*, 2016 ▸) using *ConQuest* (Bruno *et al.*, 2002 ▸) for the μ_2_-phenolato-1:2κ^2^*O*-dizinc(II) fragment as ligand gave 1683 hits. μ_2_-Dinuclear metal complexes with the quinolin-8-olato-1:2κ^2^*O* fragment gave 843 hits and among those, 83 hits for μ_2_-dinuclear Zn^II^ complexes. Of these 83 analogues, 55 structures have a μ_2_-bis­(μ_2_-quinolin-8-olato-2κ*N*;1:2κ^2^*O*)-dizinc(II) fragment containing two quinolin-8-olato moieties. Among these 55 analogues, four structures are μ_2_-dinuclear zinc(II) complexes containing two quinolin-8-olato moieties and a dpa unit. Of the four analogues, two structures are polymorphs of the Zn^II^:ligand = 2:2 dinuclear complex with the ligand in which the Cl atom of HClqdpa is replaced with an H atom, bis­(μ_2_-7-({bis­[(pyridin-2-yl)meth­yl]amino}­meth­yl)quinolin-8-olato-2κ*N*;1:2κ^2^*O*)di­zinc(II) bis­(tetra­phenyl­borate) (FEDTUH and FEDTOB; Kong *et al.*, 2022 ▸), and other two structures are the Zn^II^:ligand = 2:2 dinuclear μ_2_-type complexes with two 2-{[(pyridin-2-yl)meth­yl]amino}­meth­yl)quinolin-8-olato-2κ*N*;1:2κ^2^*O*) fragments (RIZROI; Xue *et al.*, 2008 ▸; CIGJAF; Royzen & Canary, 2013 ▸). All of the four μ_2_-dinuclear Zn^II^ complexes contain qunolin-8-olato and dpa moieties and have distorted octa­hedral geometries.

## 5. Synthesis and crystallization

The HClqdpa ligand was prepared by the reported method (Kubono *et al.*, 2015 ▸). Zinc(II) perchlorate hexa­hydrate (93.1 mg, 0.25 mmol) was dissolved in 20 mL of hot aceto­nitrile. Then a solution of HClqdpa (97.7 mg, 0.25 mmol) in 15 mL of hot aceto­nitrile was added to the zinc salt solution. The mixture was stirred for 20 min at 333 K. After slow evaporation of the solvent at room temperature in the air for one week, yellow crystals of the title compound were obtained (yield 28.2%). Analysis calculated for C_46_H_39_Cl_4_N_9_O_10_Zn_2_: C 48.02, H 3.42, N 10.96%; found: C 48.00, H 3.43, N 10.76%.

## 6. Refinement

Crystal data, data collection and structure refinement details are summarized in Table 3 ▸. All H atoms bound to carbon were positioned geometrically and refined using a riding model, with C—H = 0.95–0.99 Å and *U*_iso_(H) = 1.2*U*_eq_(C). The perchlorate ion is disordered over two sets of sites with refined occupancies of 0.510 (4) and 0.490 (4). The solvate aceto­nitrile mol­ecules are disordered within the cavities around a center of inversion, which is located in the middle of the methyl groups of the two aceto­nitrile mol­ecules. Therefore, all the atoms of aceto­nitrile were refined with 0.5 occupancy.

## Supplementary Material

Crystal structure: contains datablock(s) I. DOI: 10.1107/S2056989025010680/jp2022sup1.cif

Structure factors: contains datablock(s) I. DOI: 10.1107/S2056989025010680/jp2022Isup2.hkl

CCDC reference: 2512101

Additional supporting information:  crystallographic information; 3D view; checkCIF report

## Figures and Tables

**Table 1:** Selected geometric parameters (Å, °)

Zn1—O4	2.0496 (13)	Zn1—N10	2.3072 (16)
Zn1—O4^i^	2.0906 (12)	Zn1—N11	2.1171 (15)
Zn1—N9^i^	2.2527 (16)	Zn1—N12	2.0868 (15)
			
O4—Zn1—O4^i^	75.08 (5)	N9^i^—Zn1—N10	125.53 (6)
O4^i^—Zn1—N9^i^	74.58 (5)	N11—Zn1—N9^i^	83.56 (5)
O4—Zn1—N9^i^	145.63 (5)	N11—Zn1—N10	74.51 (6)
O4—Zn1—N10	87.06 (5)	N12—Zn1—O4^i^	97.09 (5)
O4^i^—Zn1—N10	158.76 (5)	N12—Zn1—N9^i^	93.98 (6)
O4—Zn1—N11	97.03 (5)	N12—Zn1—N10	76.40 (6)
O4^i^—Zn1—N11	118.42 (6)	N12—Zn1—N11	141.96 (6)
O4—Zn1—N12	105.45 (6)	Zn1—O4—Zn1^i^	104.92 (5)

Symmetry code: (i) 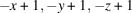.

**Table 2:** Hydrogen-bond geometry (Å, °)

*D*—H⋯*A*	*D*—H	H⋯*A*	*D*⋯*A*	*D*—H⋯*A*
C20—H20⋯O5*B*^ii^	0.95	2.44	3.124 (4)	129
C21—H21⋯O8*A*^iii^	0.95	2.43	3.152 (14)	132
C21—H21⋯O8*B*^iii^	0.95	2.30	3.094 (18)	141
C23—H23*B*⋯Cl2^iv^	0.99	2.81	3.7220 (19)	154
C26—H26⋯O5*B*^v^	0.95	2.38	3.140 (5)	137
C33—H33⋯O8*A*^vi^	0.95	2.52	3.453 (15)	168
C33—H33⋯O8*B*^vi^	0.95	2.60	3.504 (19)	160
C34—H34⋯O7*A*^vii^	0.95	2.49	3.334 (5)	148

Symmetry codes: (ii) 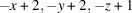; (iii) 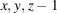; (iv) 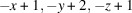; (v) 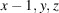; (vi) 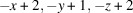; (vii) 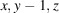.

**Table 3:** Experimental details

Crystal data	
Chemical formula	[Zn_2_(C_22_H_18_ClN_4_O)_2_](ClO_4_)_2_·C_2_H_3_N
*M*_r_	1150.44
Crystal system, space group	Triclinic, *P* 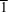
Temperature (K)	100
*a*, *b*, *c* (Å)	9.5378 (1), 10.3401 (2), 12.5859 (2)
α, β, γ (°)	68.719 (2), 89.599 (1), 86.872 (1)
*V* (Å^3^)	1154.77 (4)
*Z*	1
Radiation type	Cu *K*α
μ (mm^−1^)	4.01
Crystal size (mm)	0.21 × 0.16 × 0.07

Data collection	
Diffractometer	XtaLAB Synergy, Dualflex, HyPix
Absorption correction	Multi-scan (*CrysAlis PRO*; Rigaku OD, 2023 ▸)
*T*_min_, *T*_max_	0.784, 1.000
No. of measured, independent and observed [*I* > 2σ(*I*)] reflections	14973, 4562, 4315
*R*_int_	0.030
(sin θ/λ)_max_ (Å^−1^)	0.631

Refinement	
*R*[*F*^2^ > 2σ(*F*^2^)], *wR*(*F*^2^), *S*	0.029, 0.074, 1.05
No. of reflections	4562
No. of parameters	381
H-atom treatment	H-atom parameters constrained
Δρ_max_, Δρ_min_ (e Å^−3^)	0.30, −0.43

Computer programs: *CrysAlis PRO* (Rigaku OD, 2023 ▸), *SHELXT2018/2* (Sheldrick, 2015*a* ▸), *SHELXL2018/3* (Sheldrick, 2015*b* ▸), *PLATON* (Spek, 2020 ▸) and *OLEX2* (Dolomanov *et al.*, 2009 ▸).

## supplementary crystallographic information

### Bis[µ2-7-({bis[(pyridin-2-yl)methyl]amino-κ3N,N',N''}methyl)-5-chloroquinolin-8-olato-κ2N,O]dizinc(II) bis(perchlorate) acetonitrile monosolvate Crystal data

**Table:**

[Zn_2_(C_22_H_18_ClN_4_O)_2_](ClO_4_)_2_·C_2_H_3_N	*Z* = 1
*M**_r_* = 1150.44	*F*(000) = 586
Triclinic, *P*1	*D*_x_ = 1.654 Mg m^−^^3^
*a* = 9.5378 (1) Å	Cu *K*α radiation, λ = 1.54184 Å
*b* = 10.3401 (2) Å	Cell parameters from 10658 reflections
*c* = 12.5859 (2) Å	θ = 3.8–76.3°
α = 68.719 (2)°	µ = 4.01 mm^−^^1^
β = 89.599 (1)°	*T* = 100 K
γ = 86.872 (1)°	Block, yellow
*V* = 1154.77 (4) Å^3^	0.21 × 0.16 × 0.07 mm

### Bis[µ2-7-({bis[(pyridin-2-yl)methyl]amino-κ3N,N',N''}methyl)-5-chloroquinolin-8-olato-κ2N,O]dizinc(II) bis(perchlorate) acetonitrile monosolvate Data collection

**Table:**

XtaLAB Synergy, Dualflex, HyPix diffractometer	4562 independent reflections
Radiation source: micro-focus sealed X-ray tube, PhotonJet (Cu) X-ray Source	4315 reflections with *I* > 2σ(*I*)
Mirror monochromator	*R*_int_ = 0.030
Detector resolution: 10.0000 pixels mm^-1^	θ_max_ = 76.7°, θ_min_ = 3.8°
ω scans	*h* = −12→10
Absorption correction: multi-scan (CrysAlis PRO; Rigaku OD, 2023)	*k* = −13→11
*T*_min_ = 0.784, *T*_max_ = 1.000	*l* = −15→15
14973 measured reflections	

### Bis[µ2-7-({bis[(pyridin-2-yl)methyl]amino-κ3N,N',N''}methyl)-5-chloroquinolin-8-olato-κ2N,O]dizinc(II) bis(perchlorate) acetonitrile monosolvate Refinement

**Table:**

Refinement on *F*^2^	0 restraints
Least-squares matrix: full	Hydrogen site location: inferred from neighbouring sites
*R*[*F*^2^ > 2σ(*F*^2^)] = 0.029	H-atom parameters constrained
*wR*(*F*^2^) = 0.074	*w* = 1/[σ^2^(*F*_o_^2^) + (0.0301*P*)^2^ + 0.9245*P*] where *P* = (*F*_o_^2^ + 2*F*_c_^2^)/3
*S* = 1.05	(Δ/σ)_max_ = 0.001
4562 reflections	Δρ_max_ = 0.30 e Å^−^^3^
381 parameters	Δρ_min_ = −0.43 e Å^−^^3^

### Bis[µ2-7-({bis[(pyridin-2-yl)methyl]amino-κ3N,N',N''}methyl)-5-chloroquinolin-8-olato-κ2N,O]dizinc(II) bis(perchlorate) acetonitrile monosolvate Special details

**Table:**

Geometry. All esds (except the esd in the dihedral angle between two l.s. planes) are estimated using the full covariance matrix. The cell esds are taken into account individually in the estimation of esds in distances, angles and torsion angles; correlations between esds in cell parameters are only used when they are defined by crystal symmetry. An approximate (isotropic) treatment of cell esds is used for estimating esds involving l.s. planes.

### Bis[µ2-7-({bis[(pyridin-2-yl)methyl]amino-κ3N,N',N''}methyl)-5-chloroquinolin-8-olato-κ2N,O]dizinc(II) bis(perchlorate) acetonitrile monosolvate Fractional atomic coordinates and isotropic or equivalent isotropic displacement parameters (Å^2^)

**Table:**

	*x*	*y*	*z*	*U*_iso_*/*U*_eq_	Occ. (<1)
Zn1	0.44190 (2)	0.44836 (2)	0.63109 (2)	0.01800 (8)	
Cl2	0.82721 (5)	1.08836 (5)	0.51578 (4)	0.03096 (12)	
O4	0.51495 (13)	0.61840 (13)	0.50446 (10)	0.0205 (3)	
N9	0.67111 (15)	0.74922 (16)	0.32875 (13)	0.0213 (3)	
N10	0.46481 (16)	0.56888 (18)	0.75191 (13)	0.0241 (3)	
N11	0.23854 (15)	0.53586 (15)	0.64085 (14)	0.0209 (3)	
N12	0.61760 (15)	0.34916 (17)	0.73133 (13)	0.0220 (3)	
C14	0.57977 (17)	0.72556 (19)	0.51062 (15)	0.0196 (4)	
C15	0.56558 (18)	0.7749 (2)	0.59955 (16)	0.0223 (4)	
C16	0.64405 (19)	0.8878 (2)	0.59817 (17)	0.0237 (4)	
H16	0.636216	0.919359	0.660155	0.028*	
C17	0.73055 (19)	0.95240 (19)	0.51050 (17)	0.0238 (4)	
C18	0.74269 (18)	0.91270 (19)	0.41431 (16)	0.0224 (4)	
C19	0.8227 (2)	0.9784 (2)	0.31619 (18)	0.0273 (4)	
H19	0.875610	1.055540	0.311331	0.033*	
C20	0.8232 (2)	0.9302 (2)	0.22865 (18)	0.0294 (4)	
H20	0.874732	0.974817	0.161600	0.035*	
C21	0.7469 (2)	0.8139 (2)	0.23853 (17)	0.0267 (4)	
H21	0.749874	0.780306	0.177580	0.032*	
C22	0.66709 (18)	0.79726 (19)	0.41666 (15)	0.0198 (4)	
C23	0.45798 (18)	0.7218 (2)	0.69141 (16)	0.0229 (4)	
H23A	0.469910	0.766290	0.748177	0.027*	
H23B	0.363252	0.751390	0.656580	0.027*	
C24	0.3361 (2)	0.5285 (2)	0.81917 (16)	0.0300 (4)	
H24A	0.322200	0.583104	0.869156	0.036*	
H24B	0.345115	0.428747	0.867946	0.036*	
C25	0.21211 (19)	0.55545 (19)	0.73906 (17)	0.0251 (4)	
C26	0.0812 (2)	0.6044 (2)	0.7617 (2)	0.0325 (5)	
H26	0.063648	0.614489	0.832788	0.039*	
C27	−0.0227 (2)	0.6382 (2)	0.6793 (2)	0.0402 (6)	
H27	−0.113275	0.671490	0.692897	0.048*	
C28	0.0060 (2)	0.6232 (2)	0.5767 (2)	0.0385 (6)	
H28	−0.063362	0.649069	0.517775	0.046*	
C29	0.13779 (19)	0.56963 (19)	0.56083 (19)	0.0270 (4)	
H29	0.156804	0.556661	0.491059	0.032*	
C30	0.5936 (2)	0.5221 (3)	0.82249 (18)	0.0354 (5)	
H30A	0.569083	0.497844	0.903730	0.042*	
H30B	0.657736	0.599386	0.802052	0.042*	
C31	0.6687 (2)	0.3976 (2)	0.80709 (16)	0.0281 (4)	
C32	0.7909 (2)	0.3389 (3)	0.87016 (19)	0.0394 (6)	
H32	0.826233	0.375608	0.923078	0.047*	
C33	0.8593 (2)	0.2268 (3)	0.85444 (19)	0.0391 (5)	
H33	0.942442	0.184774	0.896895	0.047*	
C34	0.8063 (2)	0.1755 (2)	0.77627 (19)	0.0337 (5)	
H34	0.852112	0.098118	0.764251	0.040*	
C35	0.6853 (2)	0.2395 (2)	0.71628 (17)	0.0274 (4)	
H35	0.648557	0.204903	0.662453	0.033*	
Cl3A	0.8138 (3)	0.78193 (16)	0.92173 (13)	0.0249 (3)	0.510 (4)
O5A	0.6650 (3)	0.7740 (4)	0.9126 (3)	0.0470 (10)	0.510 (4)
O6A	0.8777 (15)	0.6501 (12)	0.9871 (11)	0.042 (2)	0.510 (4)
O7A	0.8706 (5)	0.8426 (4)	0.8104 (3)	0.0343 (9)	0.510 (4)
O8A	0.8358 (16)	0.8775 (11)	0.9836 (12)	0.0303 (15)	0.510 (4)
Cl3B	0.8733 (3)	0.77656 (16)	0.92142 (13)	0.0232 (4)	0.490 (4)
O5B	1.0194 (3)	0.8006 (4)	0.8992 (3)	0.0378 (9)	0.490 (4)
O6B	0.8578 (14)	0.6262 (14)	0.9709 (12)	0.046 (3)	0.490 (4)
O7B	0.7967 (6)	0.8170 (5)	0.8165 (4)	0.0442 (11)	0.490 (4)
O8B	0.823 (2)	0.8425 (13)	0.9919 (15)	0.060 (4)	0.490 (4)
N13	0.4660 (5)	0.7646 (5)	0.9584 (4)	0.0469 (10)	0.5
C36	0.4847 (5)	0.8680 (5)	0.9686 (4)	0.0355 (10)	0.5
C37	0.506 (3)	0.995 (2)	0.9846 (15)	0.044 (3)	0.5
H37A	0.427448	1.016460	1.027142	0.053*	0.5
H37B	0.511833	1.070243	0.910211	0.053*	0.5
H37C	0.593765	0.985752	1.027586	0.053*	0.5

### Bis[µ2-7-({bis[(pyridin-2-yl)methyl]amino-κ3N,N',N''}methyl)-5-chloroquinolin-8-olato-κ2N,O]dizinc(II) bis(perchlorate) acetonitrile monosolvate Atomic displacement parameters (Å^2^)

**Table:**

	*U*^11^	*U*^22^	*U*^33^	*U*^12^	*U*^13^	*U*^23^
Zn1	0.01497 (12)	0.02499 (14)	0.01315 (13)	0.00192 (9)	0.00124 (8)	−0.00632 (10)
Cl2	0.0342 (3)	0.0205 (2)	0.0395 (3)	−0.00082 (18)	−0.0051 (2)	−0.0125 (2)
O4	0.0198 (6)	0.0270 (7)	0.0164 (6)	−0.0034 (5)	0.0026 (5)	−0.0096 (5)
N9	0.0203 (7)	0.0231 (8)	0.0196 (8)	0.0030 (6)	0.0024 (6)	−0.0074 (6)
N10	0.0192 (7)	0.0379 (9)	0.0159 (7)	0.0078 (6)	−0.0014 (6)	−0.0121 (7)
N11	0.0156 (7)	0.0180 (7)	0.0280 (8)	−0.0018 (5)	0.0044 (6)	−0.0070 (6)
N12	0.0180 (7)	0.0296 (8)	0.0154 (7)	0.0026 (6)	0.0002 (6)	−0.0053 (6)
C14	0.0153 (8)	0.0245 (9)	0.0201 (9)	0.0018 (7)	−0.0014 (7)	−0.0098 (7)
C15	0.0155 (8)	0.0320 (10)	0.0221 (9)	0.0018 (7)	−0.0015 (7)	−0.0136 (8)
C16	0.0193 (8)	0.0291 (10)	0.0270 (10)	0.0063 (7)	−0.0052 (7)	−0.0164 (8)
C17	0.0195 (8)	0.0220 (9)	0.0313 (10)	0.0037 (7)	−0.0055 (7)	−0.0121 (8)
C18	0.0176 (8)	0.0199 (9)	0.0276 (10)	0.0046 (7)	−0.0016 (7)	−0.0069 (7)
C19	0.0248 (9)	0.0192 (9)	0.0333 (11)	0.0021 (7)	0.0021 (8)	−0.0046 (8)
C20	0.0298 (10)	0.0245 (10)	0.0288 (11)	0.0011 (8)	0.0083 (8)	−0.0040 (8)
C21	0.0278 (10)	0.0278 (10)	0.0224 (10)	0.0028 (8)	0.0055 (8)	−0.0074 (8)
C22	0.0158 (8)	0.0225 (9)	0.0206 (9)	0.0045 (6)	−0.0009 (7)	−0.0080 (7)
C23	0.0180 (8)	0.0339 (10)	0.0225 (9)	0.0020 (7)	0.0007 (7)	−0.0175 (8)
C24	0.0325 (10)	0.0375 (11)	0.0162 (9)	0.0063 (8)	0.0086 (8)	−0.0064 (8)
C25	0.0220 (9)	0.0215 (9)	0.0290 (10)	−0.0026 (7)	0.0113 (7)	−0.0059 (8)
C26	0.0249 (10)	0.0245 (10)	0.0492 (13)	−0.0045 (8)	0.0162 (9)	−0.0147 (9)
C27	0.0179 (9)	0.0286 (11)	0.0830 (19)	−0.0034 (8)	0.0096 (10)	−0.0309 (12)
C28	0.0193 (9)	0.0282 (11)	0.0761 (17)	0.0017 (8)	−0.0124 (10)	−0.0288 (11)
C29	0.0191 (9)	0.0210 (9)	0.0443 (12)	−0.0022 (7)	−0.0061 (8)	−0.0157 (9)
C30	0.0304 (10)	0.0594 (14)	0.0207 (10)	0.0179 (10)	−0.0109 (8)	−0.0224 (10)
C31	0.0216 (9)	0.0471 (12)	0.0149 (9)	0.0085 (8)	−0.0013 (7)	−0.0117 (9)
C32	0.0286 (11)	0.0679 (16)	0.0256 (11)	0.0181 (10)	−0.0106 (8)	−0.0244 (11)
C33	0.0269 (10)	0.0593 (15)	0.0286 (11)	0.0180 (10)	−0.0082 (8)	−0.0157 (11)
C34	0.0290 (10)	0.0366 (11)	0.0327 (11)	0.0102 (9)	−0.0033 (9)	−0.0108 (9)
C35	0.0248 (9)	0.0278 (10)	0.0272 (10)	0.0030 (8)	−0.0026 (8)	−0.0075 (8)
Cl3A	0.0257 (8)	0.0275 (5)	0.0188 (5)	0.0039 (6)	0.0036 (6)	−0.0059 (4)
O5A	0.0270 (16)	0.050 (2)	0.075 (3)	−0.0046 (14)	0.0034 (15)	−0.0351 (19)
O6A	0.059 (5)	0.021 (4)	0.035 (3)	0.007 (3)	0.000 (2)	−0.001 (2)
O7A	0.058 (3)	0.0267 (17)	0.0173 (15)	0.0030 (17)	0.0106 (18)	−0.0082 (13)
O8A	0.040 (2)	0.034 (3)	0.023 (2)	0.004 (2)	−0.0067 (17)	−0.018 (2)
Cl3B	0.0322 (10)	0.0235 (5)	0.0135 (5)	0.0042 (7)	−0.0016 (7)	−0.0070 (4)
O5B	0.0415 (18)	0.049 (2)	0.0325 (18)	−0.0244 (15)	0.0132 (13)	−0.0238 (15)
O6B	0.044 (4)	0.027 (4)	0.053 (6)	−0.002 (3)	0.022 (4)	0.000 (3)
O7B	0.066 (3)	0.044 (2)	0.025 (2)	0.003 (2)	−0.020 (2)	−0.0159 (17)
O8B	0.070 (7)	0.086 (9)	0.049 (7)	0.030 (6)	−0.018 (5)	−0.058 (7)
N13	0.057 (3)	0.043 (2)	0.041 (2)	−0.008 (2)	0.018 (2)	−0.0147 (19)
C36	0.037 (2)	0.039 (3)	0.025 (2)	0.0038 (19)	0.0072 (17)	−0.0066 (19)
C37	0.039 (4)	0.039 (3)	0.062 (8)	0.014 (3)	−0.008 (7)	−0.029 (5)

### Bis[µ2-7-({bis[(pyridin-2-yl)methyl]amino-κ3N,N',N''}methyl)-5-chloroquinolin-8-olato-κ2N,O]dizinc(II) bis(perchlorate) acetonitrile monosolvate Geometric parameters (Å, º)

**Table:**

Zn1—O4	2.0496 (13)	C24—H24B	0.9900
Zn1—O4^i^	2.0906 (12)	C24—C25	1.506 (3)
Zn1—N9^i^	2.2527 (16)	C25—C26	1.389 (3)
Zn1—N10	2.3072 (16)	C26—H26	0.9500
Zn1—N11	2.1171 (15)	C26—C27	1.376 (3)
Zn1—N12	2.0868 (15)	C27—H27	0.9500
Cl2—C17	1.7439 (19)	C27—C28	1.379 (4)
O4—C14	1.324 (2)	C28—H28	0.9500
N9—C21	1.323 (2)	C28—C29	1.388 (3)
N9—C22	1.367 (2)	C29—H29	0.9500
N10—C23	1.483 (3)	C30—H30A	0.9900
N10—C24	1.478 (2)	C30—H30B	0.9900
N10—C30	1.474 (2)	C30—C31	1.512 (3)
N11—C25	1.343 (3)	C31—C32	1.394 (3)
N11—C29	1.335 (2)	C32—H32	0.9500
N12—C31	1.334 (3)	C32—C33	1.375 (3)
N12—C35	1.347 (3)	C33—H33	0.9500
C14—C15	1.392 (3)	C33—C34	1.386 (3)
C14—C22	1.435 (2)	C34—H34	0.9500
C15—C16	1.415 (3)	C34—C35	1.380 (3)
C15—C23	1.508 (3)	C35—H35	0.9500
C16—H16	0.9500	Cl3A—O5A	1.433 (4)
C16—C17	1.363 (3)	Cl3A—O6A	1.417 (14)
C17—C18	1.415 (3)	Cl3A—O7A	1.428 (4)
C18—C19	1.415 (3)	Cl3A—O8A	1.487 (13)
C18—C22	1.419 (3)	Cl3B—O5B	1.436 (4)
C19—H19	0.9500	Cl3B—O6B	1.465 (13)
C19—C20	1.364 (3)	Cl3B—O7B	1.425 (4)
C20—H20	0.9500	Cl3B—O8B	1.369 (18)
C20—C21	1.406 (3)	N13—C36	1.145 (6)
C21—H21	0.9500	C36—C37	1.425 (19)
C23—H23A	0.9900	C37—H37A	0.9800
C23—H23B	0.9900	C37—H37B	0.9800
C24—H24A	0.9900	C37—H37C	0.9800
			
O4—Zn1—O4^i^	75.08 (5)	C15—C23—H23B	108.5
O4^i^—Zn1—N9^i^	74.58 (5)	H23A—C23—H23B	107.5
O4—Zn1—N9^i^	145.63 (5)	N10—C24—H24A	109.9
O4—Zn1—N10	87.06 (5)	N10—C24—H24B	109.9
O4^i^—Zn1—N10	158.76 (5)	N10—C24—C25	109.15 (15)
O4—Zn1—N11	97.03 (5)	H24A—C24—H24B	108.3
O4^i^—Zn1—N11	118.42 (6)	C25—C24—H24A	109.9
O4—Zn1—N12	105.45 (6)	C25—C24—H24B	109.9
N9^i^—Zn1—N10	125.53 (6)	N11—C25—C24	115.36 (16)
N11—Zn1—N9^i^	83.56 (5)	N11—C25—C26	121.91 (19)
N11—Zn1—N10	74.51 (6)	C26—C25—C24	122.64 (19)
N12—Zn1—O4^i^	97.09 (5)	C25—C26—H26	120.6
N12—Zn1—N9^i^	93.98 (6)	C27—C26—C25	118.8 (2)
N12—Zn1—N10	76.40 (6)	C27—C26—H26	120.6
N12—Zn1—N11	141.96 (6)	C26—C27—H27	120.3
Zn1—O4—Zn1^i^	104.92 (5)	C26—C27—C28	119.33 (19)
C14—O4—Zn1^i^	119.28 (11)	C28—C27—H27	120.3
C14—O4—Zn1	130.39 (11)	C27—C28—H28	120.5
C21—N9—Zn1^i^	128.74 (13)	C27—C28—C29	119.0 (2)
C21—N9—C22	118.53 (16)	C29—C28—H28	120.5
C22—N9—Zn1^i^	112.47 (12)	N11—C29—C28	121.8 (2)
C23—N10—Zn1	113.04 (11)	N11—C29—H29	119.1
C24—N10—Zn1	99.31 (12)	C28—C29—H29	119.1
C24—N10—C23	109.22 (14)	N10—C30—H30A	109.1
C30—N10—Zn1	111.46 (12)	N10—C30—H30B	109.1
C30—N10—C23	110.81 (16)	N10—C30—C31	112.34 (17)
C30—N10—C24	112.52 (16)	H30A—C30—H30B	107.9
C25—N11—Zn1	114.70 (12)	C31—C30—H30A	109.1
C29—N11—Zn1	126.21 (13)	C31—C30—H30B	109.1
C29—N11—C25	119.08 (16)	N12—C31—C30	118.83 (16)
C31—N12—Zn1	120.55 (13)	N12—C31—C32	121.85 (19)
C31—N12—C35	118.97 (16)	C32—C31—C30	119.31 (19)
C35—N12—Zn1	120.39 (13)	C31—C32—H32	120.6
O4—C14—C15	124.62 (16)	C33—C32—C31	118.8 (2)
O4—C14—C22	116.93 (15)	C33—C32—H32	120.6
C15—C14—C22	118.42 (16)	C32—C33—H33	120.2
C14—C15—C16	119.30 (17)	C32—C33—C34	119.57 (19)
C14—C15—C23	121.93 (16)	C34—C33—H33	120.2
C16—C15—C23	118.47 (16)	C33—C34—H34	120.8
C15—C16—H16	119.0	C35—C34—C33	118.4 (2)
C17—C16—C15	121.90 (17)	C35—C34—H34	120.8
C17—C16—H16	119.0	N12—C35—C34	122.35 (19)
C16—C17—Cl2	119.50 (15)	N12—C35—H35	118.8
C16—C17—C18	121.44 (17)	C34—C35—H35	118.8
C18—C17—Cl2	119.05 (15)	O5A—Cl3A—O8A	106.9 (6)
C17—C18—C19	125.74 (18)	O6A—Cl3A—O5A	110.8 (6)
C17—C18—C22	116.81 (17)	O6A—Cl3A—O7A	113.9 (6)
C19—C18—C22	117.44 (17)	O6A—Cl3A—O8A	107.9 (7)
C18—C19—H19	120.2	O7A—Cl3A—O5A	109.5 (3)
C20—C19—C18	119.51 (18)	O7A—Cl3A—O8A	107.7 (5)
C20—C19—H19	120.2	O5B—Cl3B—O6B	108.5 (6)
C19—C20—H20	120.3	O7B—Cl3B—O5B	109.9 (3)
C19—C20—C21	119.41 (18)	O7B—Cl3B—O6B	102.9 (6)
C21—C20—H20	120.3	O8B—Cl3B—O5B	110.1 (7)
N9—C21—C20	123.04 (19)	O8B—Cl3B—O6B	112.0 (7)
N9—C21—H21	118.5	O8B—Cl3B—O7B	113.2 (8)
C20—C21—H21	118.5	N13—C36—C37	178.3 (9)
N9—C22—C14	116.00 (16)	C36—C37—H37A	109.5
N9—C22—C18	122.04 (16)	C36—C37—H37B	109.5
C18—C22—C14	121.95 (16)	C36—C37—H37C	109.5
N10—C23—C15	115.07 (15)	H37A—C37—H37B	109.5
N10—C23—H23A	108.5	H37A—C37—H37C	109.5
N10—C23—H23B	108.5	H37B—C37—H37C	109.5
C15—C23—H23A	108.5		
			
Zn1—O4—C14—C15	−26.6 (2)	C16—C17—C18—C22	−3.6 (3)
Zn1^i^—O4—C14—C15	−176.44 (13)	C17—C18—C19—C20	−179.01 (18)
Zn1^i^—O4—C14—C22	5.5 (2)	C17—C18—C22—N9	−179.81 (16)
Zn1—O4—C14—C22	155.34 (12)	C17—C18—C22—C14	1.4 (3)
Zn1^i^—N9—C21—C20	−173.83 (14)	C18—C19—C20—C21	−1.5 (3)
Zn1^i^—N9—C22—C14	−7.22 (19)	C19—C18—C22—N9	0.6 (3)
Zn1^i^—N9—C22—C18	173.88 (13)	C19—C18—C22—C14	−178.28 (16)
Zn1—N10—C23—C15	−57.44 (17)	C19—C20—C21—N9	1.3 (3)
Zn1—N10—C24—C25	−51.37 (16)	C21—N9—C22—C14	178.16 (16)
Zn1—N10—C30—C31	−6.3 (2)	C21—N9—C22—C18	−0.7 (3)
Zn1—N11—C25—C24	7.1 (2)	C22—N9—C21—C20	−0.2 (3)
Zn1—N11—C25—C26	−176.26 (14)	C22—C14—C15—C16	−4.0 (3)
Zn1—N11—C29—C28	178.20 (14)	C22—C14—C15—C23	169.52 (16)
Zn1—N12—C31—C30	2.5 (3)	C22—C18—C19—C20	0.6 (3)
Zn1—N12—C31—C32	−175.94 (17)	C23—N10—C24—C25	67.1 (2)
Zn1—N12—C35—C34	176.39 (16)	C23—N10—C30—C31	−133.10 (18)
Cl2—C17—C18—C19	−3.3 (3)	C23—C15—C16—C17	−171.88 (17)
Cl2—C17—C18—C22	177.07 (13)	C24—N10—C23—C15	−166.98 (15)
O4—C14—C15—C16	177.98 (16)	C24—N10—C30—C31	104.3 (2)
O4—C14—C15—C23	−8.5 (3)	C24—C25—C26—C27	174.15 (19)
O4—C14—C22—N9	1.7 (2)	C25—N11—C29—C28	−0.6 (3)
O4—C14—C22—C18	−179.40 (15)	C25—C26—C27—C28	−0.3 (3)
N10—C24—C25—N11	34.0 (2)	C26—C27—C28—C29	2.3 (3)
N10—C24—C25—C26	−142.65 (18)	C27—C28—C29—N11	−1.8 (3)
N10—C30—C31—N12	3.0 (3)	C29—N11—C25—C24	−173.92 (17)
N10—C30—C31—C32	−178.5 (2)	C29—N11—C25—C26	2.7 (3)
N11—C25—C26—C27	−2.2 (3)	C30—N10—C23—C15	68.51 (19)
N12—C31—C32—C33	−0.8 (4)	C30—N10—C24—C25	−169.36 (17)
C14—C15—C16—C17	1.9 (3)	C30—C31—C32—C33	−179.2 (2)
C14—C15—C23—N10	54.8 (2)	C31—N12—C35—C34	−0.2 (3)
C15—C14—C22—N9	−176.47 (16)	C31—C32—C33—C34	0.4 (4)
C15—C14—C22—C18	2.4 (3)	C32—C33—C34—C35	0.0 (4)
C15—C16—C17—Cl2	−178.58 (14)	C33—C34—C35—N12	−0.1 (3)
C15—C16—C17—C18	2.1 (3)	C35—N12—C31—C30	179.09 (19)
C16—C15—C23—N10	−131.61 (17)	C35—N12—C31—C32	0.7 (3)
C16—C17—C18—C19	175.97 (18)		

Symmetry code: (i) −*x*+1, −*y*+1, −*z*+1.

### Bis[µ2-7-({bis[(pyridin-2-yl)methyl]amino-κ3N,N',N''}methyl)-5-chloroquinolin-8-olato-κ2N,O]dizinc(II) bis(perchlorate) acetonitrile monosolvate Hydrogen-bond geometry (Å, º)

**Table:**

*D*—H···*A*	*D*—H	H···*A*	*D*···*A*	*D*—H···*A*
C20—H20···O5*B*^ii^	0.95	2.44	3.124 (4)	129
C21—H21···O8*A*^iii^	0.95	2.43	3.152 (14)	132
C21—H21···O8*B*^iii^	0.95	2.30	3.094 (18)	141
C23—H23*B*···Cl2^iv^	0.99	2.81	3.7220 (19)	154
C26—H26···O5*B*^v^	0.95	2.38	3.140 (5)	137
C33—H33···O8*A*^vi^	0.95	2.52	3.453 (15)	168
C33—H33···O8*B*^vi^	0.95	2.60	3.504 (19)	160
C34—H34···O7*A*^vii^	0.95	2.49	3.334 (5)	148

Symmetry codes: (ii) −*x*+2, −*y*+2, −*z*+1; (iii) *x*, *y*, *z*−1; (iv) −*x*+1, −*y*+2, −*z*+1; (v) *x*−1, *y*, *z*; (vi) −*x*+2, −*y*+1, −*z*+2; (vii) *x*, *y*−1, *z*.
